# Impact of glutathione on acute ischemic stroke severity and outcome: possible role of aminothiols redox status

**DOI:** 10.1080/13510002.2021.1952819

**Published:** 2021-07-08

**Authors:** Marina Yurievna Maksimova, Alexander Vladimirovich Ivanov, Edward Danielevich Virus, Ksenya Alexandrovna Nikiforova, Fatima Ramazanovna Ochtova, Ekaterina Taymurazovna Suanova, Maria Petrovna Kruglova, Mikhail Aleksanrovich Piradov, Aslan Amirkhanovich Kubatiev

**Affiliations:** a Research Center of Neurology, Moscow, Russia; b Department of Molecular and Cellular Pathophysiology, Institute of General Pathology and Pathophysiology, Moscow, Russia; c Department of Nervous Diseases, Moscow State Medical and Dental University, Moscow, Russia; d Sechenov First Moscow State Medical University (Sechenov University) of the Ministry of Health of the Russian Federation, Moscow, Russia; e Russian Medical Academy of Postdoctoral Education, Moscow, Russia

**Keywords:** Acute stroke, aminothiols‌, cysteine, ‌ischemic stroke, glutathione, homocysteine, redox status, stroke severity

## Abstract

**Objective:**

Acute brain ischemia is accompanied by a disruption of low-molecular-weight aminothiols (LMWTs) homeostasis, such as homocysteine (Hcy), cysteine (Cys), and glutathione (GSH). We investigated the redox balance of LMWTs in blood plasma and its influence on ischemic stroke severity and the functional outcome in patients with an acute period.

**Patients and methods:**

A total of 177 patients were examined. Total and reduced forms of LMWTs were determined in the first 10–24 h. Stroke severity and functional state were estimated using the National Institutes of Health Stroke Scale (NIHSS) and the modified Rankin Scale (mRs) at admission and after 21 days.

**Results:**

Patients with high levels of total Hcy (> 19 μM) showed significantly reduced redox statuses of all LMWTs. Patients with low total GSH levels (≤ 1.07 μM) were at an increased risk of higher stroke severity (NIHSS > 10) compared to patients with a total GSH level > 2.64 μM (age/gender-adjusted odds ratio: 4.69, 95% CI: 1.43–15.4).

**Discussion:**

(1) low total GSH level can be considered as a novel risk marker for the severity of acute stroke in conditions of low redox status of LMWTs and (2) high Hcy levels associated with low redox status of LMWTs.

## Introduction

Ischemic stroke (IS) is a leading cause of death and disability worldwide [1]. Behind the external uniformity of the clinical presentation of IS, heterogeneity of etiological factors influences its pathogenesis. The most significant risk factors for the development of IS are arterial hypertension, stenosis of the internal carotid arteries, heart disease, including atrial fibrillation, diabetes, lipid metabolism disorders, smoking, and hyperhomocysteinemia (HHcy) [2].

Homocysteine (Hcy) is a sulfur-containing amino acid that is formed in the interim phase of the conversion of methionine to cysteine (Cys). Cys, in turn, is the limiting substrate for the synthesis of glutathione (GSH). GSH is the main low-molecular-weight antioxidant and therefore plays an important role in the cell, regulating multiple vital functions. All these metabolites are included in the low-molecular-weight aminothiol (LMWT) system and are in an oxidized or reduced state, which is characterized by a redox status (RS, the ratio of the reduced form [*r*] of the thiol to its total content [*t*]).

Multiple studies since the end of the twentieth century showed that the risk of IS depends on tHcy levels [3]. The association between HHcy and the risk of IS is currently being intensively investigated. Clinical studies of approaches that aim at reducing the level of Hcy demonstrate contradictory results for the prevention of primary, recurrent stroke, and post-stroke rehabilitation, which still cannot be satisfactorily explained [4,5]. The prognostic value of Hcy level at admission and its impact on neurological and functional outcome at acute IS remains unclear also. A number of studies have shown a positive association of the tHcy level with the neurological deficit at admission and 7 days after IS [6–9]. In addition, an association of HHcy with a negative functional outcome 3 months after stroke has been shown [8–11]. Also, HHcy was found as a risk factor for recurrent stroke or subarachnoid hemorrhage [8,11,12]. Conversely, some publications showed no significant prognostic effect of tHcy on neurological deficits. For example, it was concluded that the clinical contribution of tHcy to the functional IS outcome in the first 3 months was negligible, despite the reliable association of these indicators [13]. In spite of the correlation of tHcy with the neurological deficit at admission, no association with the functional outcome was found after 3 and 6 months after IS [7]. In a shorter period (1 month), there was no association of Hcy level with functional outcome on the Glasgow Outcome Scale [14]. It is important to note that in most clinical studies, only the total Hcy level (tHcy) was determined and studies of Hcy effect on the levels of other LMWTs and their RS were not carried out.

It was previously demonstrated that on the first day of stroke, there is a decrease in the GSH level in the blood plasma [15]. Despite the important protective role of GSH against oxidant damage in brain ischemia is well known [16], the possibility of using plasma GSH as a marker of IS severity is poorly investigated.

Experimental studies showed that brain ischemia induces significant changes in LMWTs redox balance (decreasing of its RS and r-forms) [17,18]. However, a clinical preliminary study showed that changes in LMWTs at acute IS are more complex and further knowledge of the determining factors in the acute IS period is necessary [19].

In this paper, we studied the impact of Hcy on redox balance of LMWTs in blood plasma and LMWTs influence on IS severity (according to the National Institutes of Health Stroke Scale [NIHSS]) and the functional state (according to the modified Ranking Scale [mRs]) in patients within the acute period of IS (first 24 h and after 21 days).

## Methods

### Patients

The study included 177 patients with primary IS in the carotid artery basin who were admitted in the first 10–24 h after the development of neurological disorders. Patients were admitted to the neurology department of the Research Center of Neurology from November 2016 to May 2017. The study included patients who signed voluntary informed consent for examination and treatment as well as processing of personal data. The study protocol was developed in accordance with CONSORT 2010 recommendations and was approved by the local ethics committee of the Research Center of Neurology (Moscow, Russia). All procedures performed in the study involving human participants were in accordance with the ethical standards of our institutional research committee and with the 1964 Helsinki declaration and its later amendments or similar ethical standards.

The subtype of IS was determined according to the TOAST (Trial of ORG 10172 in Acute Stroke Treatment) classification criteria. The patients had no previous cerebrovascular events (i.e. cerebral infarct, cerebral hemorrhage, or transient ischemic attack). Information on hypertension, type 2 diabetes mellitus, and heart disease (i.e. coronary heart disease, myocardial infarction, valvular disease, and atrial fibrillation) was based on the medical history and clinical data.

The main criteria for inclusion of patients in the study were age from 45 to 80 years, primary IS in the carotid artery basin, heart attack in the carotid artery basin, verified by neuroimaging, and acute stroke period in the first 10–24 h after the onset of neurological symptoms. The criteria for exclusion from the study were refusal to participate in the study, hemorrhagic stroke, type 1 diabetes mellitus, acute myocardial infarction, chronic alcoholism, decompensated renal, hepatic, or respiratory failure, heart failure III–IV functional class, cancer, and HIV infection.

The severity of the neurological disorders was rated on the NIHSS, and the degree of disability and functional independence, on the mRs [20,21].

All patients underwent magnetic resonance (MR) imaging of the brain using the Magnetom Verio (Siemens) and Magnetom Symphony (Siemens) devices with magnetic induction values of 3, 1.5, and 1.5 T, respectively. MR angiography was performed in a 3D-TOF mode to detect the intracranial artery pathology. Brain infarction was defined as a focus of increased MR signal intensity in the T2, T2 d-f, and DWI modes with a reduced diffusion coefficient on the ADC map.

After the clinical and anamnestic data, results of the neurological examination, and neuroimaging picture were compared, the diagnosis of IS in the carotid artery basin was considered reliable.

### LMWT determination

Blood samples (1 mL) were obtained at admission. Venous blood was collected in tubes containing sodium citrate (3.8%) and centrifuged at 3000× *g* for 3 min. The plasma for the total LMWT assay was collected, frozen at –80°C, and stored until analysis. Plasma (100 μL) was added to 25 μL of 5-sulfosalicylic acid dihydrate solution (230 g/L) immediately after isolation, for reduced LMWT determination. The samples were mixed thoroughly, frozen, and stored at –80°C. The t-, r-forms, and the RS of Cys, Hcy, and GSH were determined by liquid chromatography as previously described [17].

### Statistical analysis

Data collection and primary processing (identification and integration of the chromatographic peaks) were performed in MassLynx v4.1 (Waters, Milford, Massachusetts, U.S.A.). Statistical data analysis was performed using SPSS Statistics v. 22 (IBM, Armonk, New York, U.S.A.). Quantitative indicators were described using the median and quartiles, as well as the minimum and maximum values. Nonparametric Kruskal–Wallis and Mann–Whitney tests with Holm–Bonferroni correction for multiple group comparisons, and the Spearman’s rank correlation coefficient (*ρ*) were used to compare groups on a quantitative basis. For all comparisons and tests, a two-sided critical significance level (*p*) was used. Linear regression analysis was used to identify the effect of age on NIHHS, mRs, and LMWTs. The threshold value of significant differences (*p*) here was reduced to < 0.01 due to the implementation of multiple comparisons. Logistic regression analysis was performed for crude odds ratio (OR) and age- and gender-adjusted odds ratio (AOR). A *p* < 0.05 was considered to indicate a significant difference.

## Results

### Influence of total tHcy level on LMWTs

The demographic characteristics and laboratory data of patients are presented in Table 1. There was a high frequency of risk factors (i.e. hypertension, hyperlipidemia, diabetes mellitus, and coronary artery disease) in the patient cohort. Many patients had a history of chronic cerebral ischemia. No significant association was found between the LMWTs and NIHSS or mRs.

**Table 1.** Characteristics of the patients with ischemic stroke^a^.

Characteristics	Ischemic stroke (*n*=177)
Age, years	62 [55; 68]
Gender (male/female), *n* (%)	95/82 (53.7/46.3)
Chronic cerebral ischemia, *n* (%)	76 (42.9%)
NIHSS at admission	6 [3; 10]
NIHSS at 21 d	3 [2; 7]
mRS at admission	3 [2; 4]
mRs at 21 d	2 [1; 3]
*Risk factors*	
Hypertension, *n* (%)	170 (96.0%)
DM2, *n* (%)	32(18.1%)
Duration DM2, years	10 (5–12)
Hyperlipidemia, *n* (%)	124 (70.1%)
CAD, *n* (%)	52 (29.4%)
Atrial fibrillation, *n* (%)	36 (20.3%)
Current cigarette smoking, *n* (%)	58 (32.8%)
Alcohol drinker, *n* (%)	25(14.1%)
Body Mass Index > 25 kg/m^2^, *n* (%)	120 (67.8%)
Overweight, *n* (%)	55 (45.8%)
*Laboratory findings*	
Total cholesterol, mmol/L,	6.0 (5.0–6.9)
TG, mmol/L,	1.5 (1.1–2.2)
HDL-C, mmol/L,	1.7 (1.5–2.1)
LDL-C, mmol/L,	2.4 (1.9–2.9)
Fasting glucose, mmol/L,	6.7 (6.2–8.0)
Urea, mM	6.5 (5.9–6.9)
Ht, %	41 (38–44)
aPTT, s	27.2 (25.1–29.0)
Fibrinogen, g/L	3.34 (2.97–4.17)
HGB, g/L	144 (133–156)
RBC, 10^12^/L	4.8 (4.5–5.1)
WBC, 10^9^/L	7.6 (6.1–9.1)
PLT, 10^9^/L	200 (172–234)
ESR, mm/h	15 (8–23)
*LMWTs*	
tCys, μM	322 [237; 376]
tGSH, μM	1.65 [1.07; 2.60]
tHcy, μM	13.5 [9.8; 18.9]
rCys, μM	6.53 [5.18; 7.73]
rGSH, μM	0.039 [0.024; 0.062]
rHcy, μM	0.172 [0.137; 0.256]
Cys RS, %	2.1 [1.7; 2.9]
GSH RS, %	2.1 [1.5; 4.1]
Hcy RS, %	1.3 [0.9; 2.1]

Notes: aPTT, activated partial thromboplastin time; DM2, type 2 diabetes mellitus; CAD, coronary artery disease; Cys RS, cysteine redox status; ESR, erytrocyte sedimentacion rate; Hcy RS, homocysteine redox status; HDL-C, high-density lipoprotein cholesterol; HGB, hemoglobin; Ht, hematocrit; GSH RS, glutathione redox status; LDL-C, low-dencity lipoprotein cholesterol; LMWTs, low-molecular-weight aminothiols; mRs, modified Rankin scale; NIHSS, National Institutes of Health Stroke Scale; PLT, platelets; RBC, red blood cells; rCys, reduced cysteine; rGSH, reduced glutathione; rHcy, reduced homocysteine; tCys, total cysteine; TG, triglycerides; tGSH, total glutathione; tHcy, total homocysteine; WBC, white blood cells.

^a^Data presented as range (min – max) or [1st; 3rd quartiles].

**Figure 1.**
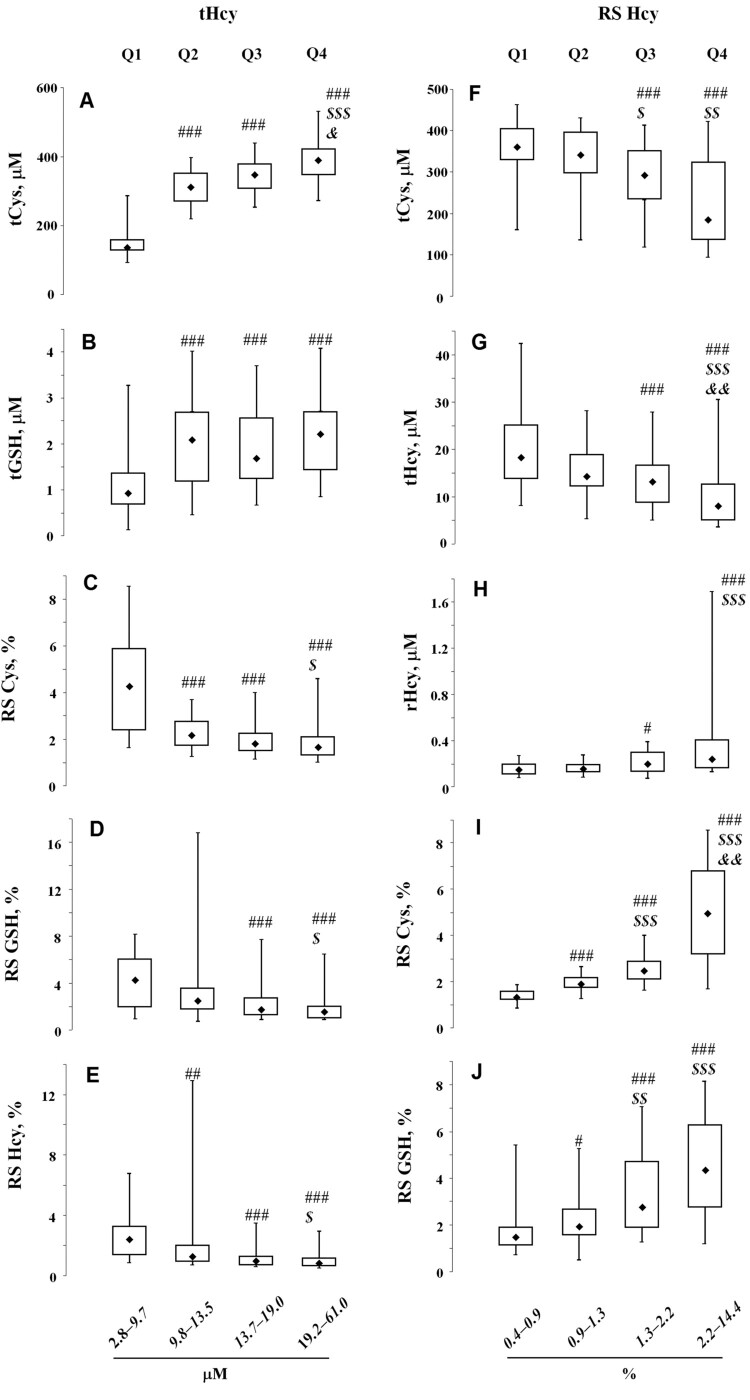
LMWTs and their RSs in patients with IS quartilized by tHcy levels (A–E) and RS Hcy (F–J). Note: ###*p* < 0.001, ##*p* < 0.01, #*p* < 0.05 compared with Q1; $$$*p* < 0.001, $$*p* < 0.01, $*p* < 0.001 compared with Q2; &&*p* < 0.01, &*p* < 0.05 compared with Q3.

Among the studied patients, the frequency of patients with HHcy (tHcy > 15 μM) was 39.5% (*N* = 70). The entire patient cohort at the first stage was divided into four equal quartiles (i.e. Q1–Q4) based on the ascending tHcy levels. As shown in Figure 1, these subgroups had significantly different tHcy and other LMWT levels. The level of total Cys (tCys) was increased throughout the quartiles Q1–Q4 (Figure 1(A)). The level of total GSH (tGSH) was significantly lower in Q1 than in the other quartiles (0.93 vs. 2.07 μM, *p* < 0.001, Figure 1(B)); however, unlike tCys, there was no tendency to an increased tGSH in Q2–Q4. The quartile Q1 was characterized by a significant increase in the RS of all thiols (Figure 1(C–E)). In the case of Hcy and Cys, the increase in their reduced forms did not compensate for the increase in their total content in Q1–Q4. Thus, in the Q4 subgroup, 68% of patients had a Hcy RS < 1.3%, while in Q1, only 19% did.

When quartilizing the cohort of patients for Hcy RS tCys and tHcy levels were tendentially reduced in the Q1–Q4 series (Figure 1(F,G)). Thus, in the Q1 subgroup, the HHcy frequency (tHcy > 15 μM) was 68.2%, while in the Q4 subgroup, it was only 14.6%. Additionally, a clear upward trend in the rHcy levels (Figure 1(H)) was indicated. Due to the close relationship of LMWT RS, the increase in Hcy RS was accompanied by an increase in RS of other thiols (Figure 1(I,J)).

### Effect of LMWTs on the neurological deficit (NIHSS) and functional outcome (mRs) at admission

Quartilizing the cohort of patients over Hcy and Cys did not reveal their influence on the risk of increased neurological deficits at admission. However, it was found that the risk of NIHSS_1_ > 10 is significantly higher in groups with low levels of tGSH or reduced GSH (rGSH) than in groups with high levels. When comparing groups of patients with tGSH ≤ 1.07 μM (Q1) and > 2.64 μM (Q4), this crude relative risk (RR) was 3.55, *p* = 0.027, the AOR 4.69, and 95% confidence interval (CI) was 1.43–15.4. The risk remained significantly increased in patients with low tGSH level (≤ 1.65 vs. > 1.65 μM), with RR = 2.07, *p* = 0.008, AOR = 2.52, and 95% CI 1.20–5.28. When comparing patients with high (> 0.039 μM) and low (≤ 0.039 μM) rGSH levels, the risk of NIHSS_1_ > 10 in the latter group was also higher: RR = 2.08, *p* = 0.011, OR = 2.61, and 95% CI 1.23–5.52. But after age and gender adjusting, significant differences in these groups were no longer observed (AOR = 1.92, 95% CI 0.93–3.96).

Further, when dividing the cohort of patients into three groups corresponding to the levels of neurological deficit (NIHSS_1_ < 6, 6–10, and > 10), the incidence of low levels of tGSH (≤1.3 μM) was noticeably higher (Table 2) in the group with NIHSS_1_ > 10. Meanwhile, no significant differences in the frequency of HHcy, low levels of tHcy, Cys RS, and Hcy RS were found.

**Table 2.** Distribution of some indicators of LMWTs depending on the degree of neurological deficit at admission in patients with acute IS.

	NIHSS_1_ < 6	NIHSS_1_ 6–10	NIHSS_1_ > 10	RR^a^	AOR^a^	95%CI
*N*	86	48	43			
Age, y	61.5 [52.5; 68]	61 [53.5; 66]	65 [58; 75]^b^			
HHcy (tHcy>15 μM)	32	23	15	0.94	0.48	0.33–1.67
Low tHcy (≤5 μM)	6	3	4	1.33	1.34	0.34–5.27
Low tGSH (≤ 1.3 μM)	26	14	24	**1.85**^c^	**3.09**	**1.39**–**6.86**
Low Cys RS (< 1.68%)	21	15	10	0.95	0.82	0.32–2.09
Low Hcy RS (≤ 1.32%)	43	21	26	1.21	1.32	0.60–2.90

Notes: Cys RS, cysteine redox status; Hcy RS, homocysteine redox status; HHcy, hyperhomocysteinemia; LMWTs, low-molecular-weight aminothiols; NIHSS, National Institutes of Health Stroke Scale; tGSH, total glutathione; tHcy, total homocysteine.

^a^ Comparison NIHSS>10 and NIHSS<6 groups.

^b^ Kruskal–Wallis test  = 0.031.

^c^ *p*=0.005.

A more detailed analysis showed that Cys RS and Hcy RS are important modulators of the association between low tGSH levels and the high risk of neurological deficits (NIHSS_1_ > 10). Table 3 shows that in the group with low levels of tGSH (≤ 1.65 μM) and Hcy RS (≤ 1.32%), there is a significantly higher frequency of NIHSS_1_ > 10 than in the group with high levels of these indicators. Also, low tGSH level was associated with higher frequency of NIHSS_1_ > 10 in patients with low Hcy RS, than patients with high Hcy RS. Of note, patients with low tGSH and high Hcy RS had, on the contrary, a lower risk of high neurological deficits than patients with low tGSH and low Hcy RS.

**Table 3.** Cooperative influence of tGSH and Hcy RS on risk of high neurological deficits (NIHSS > 10) at admission.

Group^a^	*N*_total_	*N*_NIHSS > 10_	%	RR^b^	*p*	AOR^b^	95%CI
L^tGSH^L^HcyRS^	31	16	52	–	–	–	–
L^tGSH^H^HcyRS^	55	12	22	2.36	0.0047	2.95	1.13–7.70
H^tGSH^L^HcyRS^	59	11	19	2.77	0.0013	3.47	1.23–9.78
H^tGSH^H^HcyRS^	28	3	11	4.82	0.0008	7.20	1.64–31.7

Notes: Hcy RS, homocysteine redox status; NIHSS, National Institutes of Health Stroke Scale; tGSH, total glutathione.

^a^ L^tGSH^, low tGSH (≤ 1.65 μM); H^tGSH^, high tGSH (> 1.65 μM); L^HcyRS^, low Hcy RS (≤ 1.32%); H^HcyRS^, high Hcy RS (> 1.32%).

^b^ L^tGSH^L^HcyRS^ group was compared with other groups.

Similar results were obtained when patients were divided into groups with low/high Cys RS and low/high tGSH levels (Table 4). Again, patients with low tGSH and Cys RS most frequently displayed NIHSS_1_ > 10. The strongest effect of low tGSH on the risk of neurological deficit was observed in patients with low Cys RS. In the group of patients with high Cys RS, no such association was observed. In addition, low Cys RS was associated with this risk in the group of patients with low tGSH. Also, there were no differences in the frequency of NIHSS_1_ > 10 between patients with low or high tGSH against a background of high Cys RS.

**Table 4.** Cooperative influence of tGSH and Cys RS on risk of high neurological deficits (NIHSS > 10) at admission.

Group^a^	*N*_total_	*N*_NIHSS>10_	%	RR^b^	*p*	AOR^b^	95%CI
L^tGSH^L^CysRS^	32	15	47	–	–	–	–
L^tGSH^H^CysRS^	54	13	24	1.95	0.029	3.73	1.50–9.25
H^tGSH^L^CysRS^	65	7	11	4.35	< 0.0001	6.27	2.10–18.9
H^tGSH^H^CysRS^	29	7	24	1.94	0.064	3.78	1.17–12.2

Notes: Cys RS, cysteine redox status; NIHSS, National Institutes of Health Stroke Scale; tGSH, total glutathione.

^a^ L^tGSH^, low tGSH (≤ 1.65 μM); H^tGSH^, high tGSH (> 1.65 μM); L^CysRS^, low Cys RS (≤ 2.15%); H^CysRS^, high Cys RS (> 2.15%).

^b^ L^tGSH^L^CysRS^ group was compared with other groups.

Low tGSH (≤ 1.65 μM), combined with low Cys RS (< 2.15%) remained a risk factor for NIHSS > 7 and 21 days after IS: RR = 2.19, *p* = 0.015, AOR = 2.86, and 95% CI 1.21–6.76. Also, low tGSH (≤ 1.65 μM), combined with low Hcy RS (< 1.32%) remained a risk factor for NIHSS > 7 and 21 after days IS: RR = 2.11, *p* = 0.026, AOR = 2.43, 95% CI 1.01–5.89.

We did not observe a significant negative effect of a low tGSH level (≤ 1.65 μM) alone or in combination with a low Hcy RS (≤ 1.32%) or Cys RS (≤ 2.15%) on the functional state of patients on the mRs scale at admission and after 21 days of IS.

Using linear regression analysis, we did not reveal a significant effect of the age of patients on such indicators as NIHSS and mRs both in the general cohort and among patients with low/high levels of tHcy, tGSH, Cys RS, Hcy RS. Among LMWTs, there was a weak negative effect of age on tGSH in the entire cohort of patients (slope = −0.023, *R*^2^ = 0.045, *p* = 0.007). And among patients over 62 years old, a slight negative effect of age on the level of not only tGSH, but also tCys was revealed (slope = −0.061, *R*^2^ = 0.131, *p* = 0.001 and slope = −6.52, *R*^2^ = 0.116, *p* = 0.003 correspondingly). Moreover, a slight effect of age on the tCys level was observed in groups of patients with NIHSS_21_ > 7 (slope = −4.78, *R*^2^ = 0.191, *p* = 0.008) and with mRs_21_ > 2 (slope = −3.6, *R*^2^ = 0.123, *p* = 0.004).

## Discussion

In our patient cohort, the frequency of HHcy was 39.5%, which is significantly higher than that in the general population (∼5–7%), highlighting the importance of this factor in the pathology of stroke [22]. In our study, no direct effect of Hcy on neurological deficits and 3-week IS prognosis was found upon admission, although a number of studies have found a positive association of tHcy level with NIHSS score at admission [7,23]. Obviously, the Hcy level is affected by multiple factors, preventing a clear determination of the significance of the stroke severity indicator. Our results show that although patients with low tHcy level are characterized by a lower level of tGSH than patients with normal tHcy, patients with HHcy do not experience an increase in tGSH levels, despite high levels of tCys (the rate-limiting substrate for GSH synthesis). Simultaneously, our study found an increased risk of high neurological deficits (NIHSS_1_ > 10) in patients with low levels of tGSH.

GSH acts as the main intracellular thiol, which plays an important role in the antioxidant protection and resistance of cells to oxidative stress (OS). The dramatic activation of a number of reactive oxygen species-producing enzymes was found in experimental brain ischemia, indicating the progression of generalized OS and endothelial dysfunction [24]. Although the role of GSH as a major cell antioxidant has been studied for a very long time, to the best of our knowledge, the association between plasma tGSH level and neurological deficit has not been previously described. LMWTs exist in dynamic equilibrium, their content and RS are determined both by the intensity of oxidative processes in the plasma and by the transport of reduced forms from/into the cells. The close relationship between the plasma and cerebral LMWTs pools has been shown in experimental models of cerebral ischemia. In first, a decrease in RS of LMWTs in plasma was detected in a model of acute focal and global brain ischemia, that was accompanied by a decrease in rGSH content or an increase in oxidized GSH in brain tissue [17,18]. Secondly, the ability of GSH to alleviate of brain injury when it is injected into the blood was shown in the model of transient focal cerebral ischemia [25,26]. Finally, previously, the use of an *N*-acetylcysteine significantly improved neurological and functional outcomes over 3 months in patients with acute IS [27]. The positive effect of *N*-acetylcysteine is believed to be based on the stimulation of GSH synthesis because this drug penetrates cells easily, unlike Cys. In addition, as a thiol, *N*-acetylcysteine reduces other LMWTs, thereby increasing their redox status. In this regard, it becomes clear why a low tGSH level can act as a risk marker for increased IS severity in combination with low Cys RS and Hcy RS. Although, in general, there was a negative association of Hcy RS or Cys RS with the tGSH level in its entire range, this was not observed for low tGSH concentrations.

The weak but quite significant negative effect of age on the tGSH level revealed in this work, especially in the group of older patients, is in full agreement with the results of experimental studies. Experimental studies have shown that during aging, GSH levels decrease in many tissues, especially the brain [28–30]. Age-related decrease in GSH in the brain plays a key role in the aggravation of brain infarction [31]. Interestingly, although in groups of patients with poor outcomes (mRs_21_ > 2) and increased neurological deficits (NIHSS_21_ > 7), we did not find such a pattern, but the quite noticeable negative effect of age on the level of tCys as a rate-limiting source of GSH synthesis creates the prerequisites for to limit its metabolism in elderly patients.

It should be noted that an earlier study in a small patient cohort (*N* = 19) for the late post-stroke period (1–2 years later), no changes in Cys, Hcy, or cysteinyl-glycine RS were detected compared to the control group, despite a significantly higher level of tHcy in patients [32]. A later study on the post-stroke period (1.5–3 years after stroke) confirmed these findings, but the larger sample size made it possible to show that patients with HHcy (> 15 μM) had significantly lower Hcy RS than patients without HHcy (0.9 vs. 1.4%, *p* < 0.01) [33]. At the same time, there were no differences in Cys and GSH RS in these two subgroups. In addition, a small-scale study of patients who were recently diagnosed with IS (*n* = 20) showed an increase not only in tHcy and tCys, but also in their reduced forms compared to a group of healthy volunteers [34]. Although data on the RS of LMWTs and the effect of tHcy were not evaluated, the authors did not report any evidence of changes in the LMWTs RS in patients.

It can also be assumed that an increase in the level of tCys, which is characteristic of HHcy, leads to an additional increase in the level of free Hcy (mainly in the form of mixed disulfides Hcy-S-S-Cys) due to the competitive displacement of the latter. Free Hcy, as well as amino acids, can enter endothelial cells through exchange transport systems, which increases its intracellular level. Another piece of evidence for the role of HHcy in the LMWT system imbalance was the disappearance of the positive association of Hcy RS with GSH RS in patients with HHcy in our study. Thus, this gives reason to consider HHcy not only as a factor associated with chronic OS, but also as an important factor that potentiates the development of acute OS.

## Conclusion

Our study has shown that low tGSH level can be considered a risk marker for stroke severity in the acute period. Although no influence of LMWT RS on stroke severity and the degree of disability was found, low Cys RS or Hcy RS are important factors of this association. These results demonstrate the promise of research on factors affecting GSH and therapeutic approaches to support its metabolism. At the same time, studies of GSH as a marker of the severity and prognosis of IS among patients with different subtypes of stroke are of interest. Hcy is one of the important factors that have a significant, but indirect effect on GSH metabolism. HHcy is associated with decreasing RS Hcy, as well as RS of other LMWTs. This appears to be a feature of LMWT homeostasis during acute stroke. Thus, HHcy is an important factor in LMWT homeostasis in the acute period of IS, and we conclude that Hcy potentiates the reduction in the redox balance of LMWTs and thus in the development of generalized OS. tHcy level was negatively associated with reduced LMWT RS, indicating that the influence of HHcy on stroke severity may be mediated through the disruption of the thiol-disulfide balance of the LMWT system.
